# Sigmoid volvulus in pregnancy and puerperium: a case series

**DOI:** 10.4076/1757-1626-2-9275

**Published:** 2009-09-17

**Authors:** Ali Kolusari, Mertihan Kurdoglu, Ertan Adali, Recep Yildizhan, H Guler Sahin, Cetin Kotan

**Affiliations:** 1 Department of Obstetrics and Gynecology, Yuzuncu Yil University School of Medicine, Van, Turkey; 2 Department of General Surgery, Yuzuncu Yil University School of Medicine, Van, Turkey

## Abstract

Intestinal obstruction due to sigmoid volvulus during pregnancy is rare. The presenting signs/symptoms seen in these patients are the same as with non-pregnant patients. Fetal and maternal mortality rates are higher during pregnancy due to delays in diagnosis. We aimed to present four patients diagnosed with sigmoid volvulus during pregnancy and puerperium in our clinic. Diagnosis requires a high index of suspicion in a patient who presents with complaints of abdominal pain and evidence of bowel obstruction. Prompt intervention is necessary to minimize maternal and fetal morbidity and mortality.

## Introduction

Intestinal obstruction in pregnancy is a rare but serious complication with significant maternal and fetal mortality, often due to delay in both diagnosis and treatment [1]. The incidence of intestinal obstruction in pregnancy ranges from 1 in 1500 to 1 in 66431 deliveries [2,3]. Common causes of gestational intestinal obstruction include adhesions, volvulus, intussusceptions, hernia, carcinoma and appendicitis [3]. The presenting signs/symptoms seen in these patients are the same as with non-pregnant patients and often unspecific and a high level of suspicion is essential for early diagnosis. Fetal and maternal mortality rates are higher during pregnancy due to delay in diagnosis [4]. In this report, we aimed to present four patients diagnosed with sigmoid volvulus during pregnancy and puerperium in our clinic.

## Case presentation

### Case report 1

A 40-year-old Turkish woman, gravida 11, para 10 was admitted to emergency clinic with complaints of nausea, vomiting, abdominal pain and constipation. The physical examination revealed that the patient was in good general condition with normal vital signs except for a distended and tender abdomen. Routine laboratory examination results were normal except for an elevated white blood cell count of 12.1 × 103/µL. There was no history of previous abdominal surgery and her previous pregnancies had been uncomplicated. Ultrasound showed dilated small bowel loops and a pregnancy at 7 weeks. She was taken to operation with an initial diagnosis of sigmoid volvulus and was found to have a distended sigmoid colon due to volvulus and the descending colon with necrosis. Resection of sigmoid colon and descending colon and a colo-colic anastomosis was performed. She was remained in hospital for 12 days and then allowed home. The pregnancy proceeded uneventfully and she subsequently had a normal delivery of healthy 3100 grams female infant.

### Case report 2

A 24-year-old Turkish woman, gravida 3, para 3 was referred to our hospital's emergency department with abdominal pain. She had abdominal pain and nausea for two days and the physical examination revealed a tender and mildly distended abdomen. Cervix was dilated to 5 cm and 70% effaced. Ultrasonography revealed a 31 weeks dead fetus. Routine laboratory examination results were normal except for an elevated white blood cell count of 19 × 103/µL. She had a spontaneous vaginal delivery of a 1730 grams stillborn female infant. In the postpartum period, she continued to have abdominal pain getting worse. Abdominal radiographs revealed a massive distention and air fluid levels. She was taken to operation with an initial diagnosis of acute abdomen and during operation sigmoid colon and the descending colon was seen to be massively distended and necrosed due to volvulus. Resection of sigmoid colon and descending colon and proximal colostomy was performed. Colostomy was closed after three months and colorectal anastomosis was done.

### Case report 3

A 21-year-old Turkish woman, gravida 1 was referred to our hospital with a diagnosis of preterm delivery at 32 weeks of gestation. The physical examination revealed that the patient was in good general condition with normal vital signs except for a mildly distended and tender abdomen. Vaginal examination revealed a normal cervix with no dilatation or effacement. She had abdominal pain, nausea and constipation for two days. Routine laboratory examination results were normal except for an elevated white blood cell count of 15 × 10^3^/µL. Due to uterine contractions, abdominal pain and increased distention, she was taken to operation with an initial diagnosis of preterm labor and volvulus. Cesarean section was performed and a male infant weighing 1880 grams was delivered with an apgar score of 3-7. In the operation, the sigmoid colon and left colon was found to be extremely ischemic and necrosed. Resection of sigmoid colon and descending colon and proximal colostomy was performed. Colostomy was closed after three months and colorectal anastomosis was done.

### Case report 4

A 26-year-old Turkish woman, gravida 4, para 3 was admitted to our hospital's emergency department three days after delivery with a history of sudden onset lower abdominal pain, nausea, vomiting and constipation. The patient had a spontaneous vaginal delivery at home. There was no history of previous abdominal surgery and her previous pregnancies had been uncomplicated except the first one ended in abortus at eighth weeks of gestation. The physical examination revealed that the patient was in good general condition with normal vital signs except for an extremely distended and tender abdomen. Routine laboratory examination results were normal except for an elevated white blood cell count of 16.4 × 10^3^/µL. Abdominal radiographs revealed an abnormal gas pattern, with a dilated colon in the upper abdomen and air fluid levels. She was taken to operation with an initial diagnosis of sigmoid volvulus and was found to have a distended sigmoid colon and the descending colon with necrosis. Resection of sigmoid colon and descending colon and proximal colostomy was performed. In fourth postoperative day, her general condition got worse and she was transferred to intensive care unit due to aspiration pneumonia and ischemic encephalopathy. She died at 52^nd^ postoperative day due to cardiopulmonary arrest.

## Discussion

In the United States, sigmoid volvulus is usually reported in institutionalized, debilitated, or chronically constipated patients who have long redundant sigmoid colons [5]. A high incidence reported in Africa has been attributed to the high-fiber vegetable diet indigenous to that population [6].

Common causes of gestational intestinal obstruction include adhesions, volvulus, intussusceptions, hernia and appendicitis [3]. Volvulus of the sigmoid colon is the most common cause of intestinal obstruction complicating pregnancy, accounting for up to 44 per cent of cases [5]. Another study showed that in approximately 25% of cases, obstruction is the result of volvulus, usually affecting the large bowel [7]. In our cases there was no history of previous operation and there were no adhesions during operation. Obstruction was secondary to volvulus.

Sigmoid volvulus is very rare in the non-gravid female of childbearing age, and in the pregnant woman occurs most often in the third trimester. Harer and Harer surmised that this may be due to the increasing size of the uterus elevating a mobile sigmoid colon from the pelvis and producing a partial obstruction either due to pressure or kinking of the bowel [8].

The diagnosis of bowel obstruction in pregnancy is often delayed because the symptoms mimic typical pregnancy-associated complaints [4]. The classical signs of bowel strangulation such as vomiting, distention and constipation, can be diminished or even absent [9]. The most consistent signs of intestinal obstruction were abdominal pain and leukocytosis. The abdominal pain was mild and colicky in nature but became constant and severe, probably due to vascular compromise [10]. A detailed ultrasound examination may help in the differential diagnosis. Plain abdominal films demonstrate typical patterns of obstruction in 91% of the cases. At the usual dose of 0.001 Gy per film, even serial films obtained for follow up of the patients with suspected bowel obstruction, carries negligible risk for the fetus in the third trimester [5]. All of our cases had abdominal pain, nausea and leucocytosis and three had constipation.

The treatment of bowel obstruction in pregnant women is similar to that of nonpregnant women. The basis of therapy is timely surgery [4]. Surgery should be performed via midline vertical incision. In the third trimester, if adequate intestinal exposure cannot be obtained, caesarean section must be performed [11]. The entire bowel should be examined for other areas of obstruction. Bowel viability should be assessed carefully and segmental resection with or without anastomosis is often necessary [4]. Since the time from beginning of symptoms to surgery is at least 48 hours, all of our cases had ischemia and necrosis and all had resection.

When intestinal obstruction complicates pregnancy, both mother and fetus are at risk [4]. In one series of 66 pregnant patients with bowel obstruction, 23% required bowel resection with a fetal death rate of 26% and four maternal deaths [5]. In a review on intestinal obstruction during pregnancy in 1937, it has been reported that the maternal mortality rate was 21 per cent and the fetal mortality rate was 50 per cent [3]. In a brief communication by Sascha Dua, a case presented 3 days postpartum [12] was similar to our fourth case.

Sigmoid volvulus is likely to have been precipitated by colonic mobility combined with a distortion of the sigmoid colon, and also by the rapid change in uterine size following delivery [12]. Diagnosing the reasons for an acute abdomen is difficult in the immediate postpartum. Increased abdominal girth and the difficulty of eliciting abdominal signs (due to the loss of abdominal wall tone) may mask the signs of peritonism [12].

Sigmoid volvulus complicating pregnancy is an uncommon and potentially devastating problem [13]. The diagnosis of intestinal obstruction from any cause in pregnancy is made more difficult by the common overlapping complaints of nausea, vomiting and abdominal pain [14].

## Conclusion

Diagnosis of sigmoid volvulus in pregnancy requires a high index of suspicion in a patient who presents with complaints of abdominal pain and evidence of bowel obstruction. Prompt intervention is necessary to minimize maternal and fetal morbidity and mortality.

## Consent

Written informed consents were obtained from the patients for publication of these case reports. Copies of the written consents are available for review by the Editor-in-Chief of this journal.

## Competing interests

The authors declare that they have no competing interests.

## Authors' contributions

AK and HGS managed the cases and were the major contributors in writing the manuscript. They operated the cases together with CK. MK, EA and RY contributed to editing the manuscript and provided data. All the authors read and approved the final manuscript.
